# RNAi-Based Bioinsecticides for Controlling Vector-Borne Diseases

**DOI:** 10.3390/genes16111276

**Published:** 2025-10-28

**Authors:** Krystal Maya-Maldonado, Antonio Celestino-Montes, Victor Cardoso-Jaime

**Affiliations:** 1 Biochemistry Department, Center for Research and Advanced Studies, Mexico City 07360, Mexico; kmaya@cinvestav.mx; 2 Unidad de Investigación Especializada en Microbiología, Universidad Autónoma de Guerrero, Chilpancingo de los Bravo 39105, Mexico; 20611@uagro.mx; 3 Independent Researcher, Baltimore, MD 21205, USA

**Keywords:** mosquito, ticks, kissing bugs, malaria, dengue, Chagas disease, Lyme disease, dsRNA, paratransgenesis

## Abstract

Vector-borne diseases account for 17% of all infectious diseases. The most effective strategies for controlling these diseases have focused on decreasing the vector population, primarily through the use of insecticides. Many insecticides have no specific targets, harming pollinators and beneficial insects. Additionally, the vector populations are developing resistance, reducing the effectiveness of these strategies and increasing ecological damage. Double-strand RNA (dsRNA) is widely used in insects to study gene function by knocking down their expression. Recently, this technology has been applied to develop RNAi-based insecticides for controlling agricultural pests. These biopesticides demonstrate high specificity, as insects do not develop resistance to them, and they cause minimal ecological damage. These pesticides knock down the expression of key genes related to vital functions, development, and reproduction, which affects the insect life cycle and consequently decreases their populations. This review focuses on using RNA interference (RNAi)-based insecticides for controlling major insect vectors, including mosquitoes, kissing bugs, and ticks. We examine the advancements and challenges associated with this technology, considering the complex life cycles and feeding behavior of these insects. Furthermore, we discuss gaps in knowledge about vector biology and delivery strategies for dsRNA, which need to be addressed to enhance the application and efficiency of this emerging technology for controlling vector-borne diseases.

## 1. Introduction

Vector-borne diseases account for 17% of all infectious diseases and result in approximately 700,000 deaths each year. Although mosquitoes are the primary vectors of several pathogens, including *Plasmodium*, which is the deadliest parasite, and dengue virus, the most prevalent viral disease in the world, other vectors are gaining more importance nowadays, such as kissing bugs, ticks, fleas, and others [1]. Notably, the most effective strategies for controlling vector-borne diseases are focused on controlling the vector population, mainly through the use of synthetic pesticides [2,3]. However, vectors have developed insecticide resistance, and their use has serious environmental and human health impacts; this has led to a reduction in effectiveness and an increase in side effects [4,5].

In recent years, a new class of pesticides known as biopesticides has emerged. These are primarily based on plant-derived compounds, microbial agents, or nanoparticles, offering environmentally friendly alternatives to conventional chemical pesticides, since they are biodegradable, have a lower risk to human health, and target specific species. Nevertheless, biopesticides also present certain limitations, including a short shelf life and variability in the concentration of active ingredients, which often depends on the source plant batch. These factors pose challenges in achieving consistent efficacy. Additionally, multiple formulations are necessary to target a broad spectrum of insect species [6].

**Figure 1:**
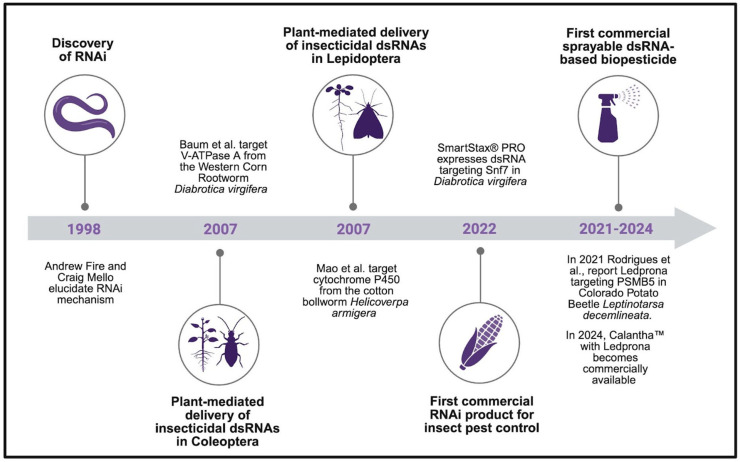
Timeline of major advances in the development of RNAi technologies for agriculture. In 1998, Fire and Mello described the RNAi mechanism in *C. elegans* [9]. In 2007, Baum et al. demonstrated plant-mediated dsRNA delivery targeting V-ATPase A in Coleoptera [11] and Mao et al. targeted cytochrome P450 gene in Lepidoptera [12]. In 2022, the first commercial RNAi product, a transgenic corn targeting the Sucrose non-fermenting 7 gene (Snf7) in Coleoptera became available [13]. In 2021, Ledprona, the first sprayable dsRNA biopesticide targeting the Proteasome subunit beta 5 (PSMB5) in the Colorado Potato Beetle, was reported. In 2024, Calantha^TM^, developed by GreenLight Biosciences and containing Ledprona, was released commercially. Created in BioRender. Maya-Maldonado, K. (2025) under agreement number CW28TU6IFQ.

RNA interference (RNAi) is a well-conserved mechanism in eukaryotes, in which small RNAs bind to complementary long RNA targets and trigger their degradation, which involves Argonaute-family nucleases, leading to reduced levels of target mRNA and gene silencing [7,8]. In the wild, the RNAi pathway modulates the expression of endogenous and exogenous genetic elements, including self-genes and invasive genes from viruses. The mechanism of RNAi was first described in *Caenorhabditis elegans* [9]. In 2006, Andrew Z. Fire and Craig C. Mello were jointly awarded the Nobel Prize in Physiology or Medicine for their discovery. Since its discovery, RNA interference (RNAi) has been widely used as a reverse genetics tool for characterizing the function of genes. More recently, it has also been explored as an innovative approach for pest management (Figure 1). In both applications, gene silencing is induced by the exogenous delivery of double-stranded RNA (dsRNA), which is processed to small interfering RNAs (siRNAs) that initiate sequence-specific degradation of target mRNAs.

RNAi-based pesticides have several advantages over other biopesticides, since RNA is a natural molecule, which means it is chemically non-toxic for any living organisms, including humans. Additionally, its specificity is exceptionally high, and cross-reactivity only occurs when species are phylogenetically very closely related [10]. Although most of the research, development, and use have been focused on managing crop pests, their application in controlling vectors of human and animal diseases has received less attention.

In this review, we discuss recent advances in RNAi-based biopesticides for controlling insect vectors. We emphasize the challenges associated with delivering dsRNA to different vector species, particularly those with complex life cycles. Additionally, we suggest strategies based on silencing previously characterized genes involved in development, reproduction, and lethality as a potential target to suppress the vector populations.

## 2. RNAi-Based Approaches in Agriculture

Historically, the impact of insects on crops has been broadly recognized, including a reduction in annual agricultural production [14]. Several strategies, such as the use of chemical pesticides, are employed to combat insect pests. However, in recent years, innovative and more sustainable approaches, such as RNAi-based biopesticides, have been developed to control agricultural pests.

One of the first studies demonstrating the potential of RNAi-based technologies for controlling insect pests was conducted in Coleoptera [11]. In this study, the authors showed that the ingestion of dsRNA induced RNA interference in several essential genes, resulting in high mortality. At the same time, another study in Lepidoptera also demonstrated that plant-mediated RNA interference of cytochrome P450 gene (*CYP6AE14*) reduces gene expression, causes effects in larval growth, and increases susceptibility to insecticides [12]. Together, these studies marked major breakthroughs in the field (Figure 1).

RNAi-based approaches have made significant advances in pest management, with some products already commercially available. In 2022, SmartStax^®^ PRO, a transgenic corn crop expressing dsRNA targeting the Sucrose non-fermenting 7 (Snf7) gene from the coleopteran *Diabrotica virgifera* [13], became the first commercial RNAi product (SmartStax^®^ PRO) to combat insect pests (Figure 1). Similarly, in 2021, was reported Ledprona, the first sprayable RNAi-based (dsRNA) biopesticide targeting the Proteasome subunit beta 5 (PSMB5) in the Colorado Potato Beetle *Leptinotarsa decemlineata* [15]. Calantha^TM^ developed by GreenLight Biosciences, containing Ledprona as active ingredient, has been approved by the United States Environmental Protection Agency (EPA) and is commercially available since 2024 [15,16] (Figure 1).

Although RNAi-based insecticides for vector control are still in the early stages of development and significant efforts are needed for their field application, this technology represents a promising approach for the control of vector-borne diseases. After all, the use of insecticides has been the most effective strategy in controlling pest insects that are important for agriculture and human health.

### Is Gene Selection the Key to Effective RNAi-Based Approaches?

Several studies have reported successful interference in vital genes for pests. For example, in *Helicoverpa armigera,* the suppression of Juvenile Hormone (JH) acid methyltransferase (JHAMT) and JH-binding protein (JHBP) has been achieved by transgenic cotton plants able to produce dsRNA targeting these genes [17]. Similarly, disruption of the expression of the β-actin gene in the Colorado potato beetle, *L. decemlineata*, causes lethality in larvae [18]. In contrast, some studies have shown that in some instances, gene interference by RNAi does not always result in lethality. A study on the brown planthopper, *Nilaparvata lugens*, demonstrated the suppression of three genes overexpressed in the insect gut, including the hexose transporter gene NlHT1, the carboxypeptidase gene Nlcar, and the trypsin-like serine protease gene Nltry, through the ingestion of transgenic rice plants producing dsRNA. However, lethality was not observed [19], indicating that gene knockdown does not always lead to a lethal phenotype. Thus, proper selection of target genes is necessary to guarantee a lethal phenotype.

In fact, all these works are based on a plant-mediated delivery strategy, which has been highly successful in delivering dsRNA to the insect due to the close association between the pest’s life cycle and plants. However, beyond successful knockdown, the lethality documented is associated with the target gene selected. For example, those that are ubiquitously expressed (i.e., expressed in several tissues) versus those that are locally expressed (i.e., expressed in the insect gut). As such, genes related to development [17] have been shown to have lethality compared to those related to digestion [19]. In the next section, we discuss several studies that employ an RNAi approach to silence genes involved in insect development and reproduction, as well as their potential application in RNAi-based insecticides targeting insect vectors.

## 3. Potential Genes for Suppression of Insect Vector Populations Using RNAi-Based Insecticides

### 3.1. Targeting Development-Related Genes

Insects undergo morphophysiological processes, such as metamorphosis, which involves several developmental changes, including molting or a complete restructuring of the body. These developmental changes make this transition a key time to block specific genes. In this section, we discuss attractive candidate genes involved in the development of various insects that have been studied, including chitin synthase and actin, as potential targets for designing RNAi strategies that enable the biological control of vectors.

#### 3.1.1. Chitin Metabolism

Chitin synthase is an enzyme that catalyzes the biosynthesis of N-acetylglucosamine to form chitin, making it the most abundant aminopolysaccharide in nature. Therefore, chitin synthase is essential for a variety of organisms, from fungi to insects [20,21,22,23]. Chitin is a key component of the exoskeleton that gives structural form to insects, whose physiological functions represent a target for new strategies to control their population [24,25]. In insects, chitin is necessary for the structuring and function of the peritrophic matrix, which divides the midgut into digestive compartments and, in turn, acts as a protective barrier against pathogens and microbial infections [26,27]. In insect pests, blocking genes related to chitin metabolism has multiple outcomes, ranging from reduced chitin production to failure of pupal ecdysis or adult emergence [28,29,30].

**Figure 2:**
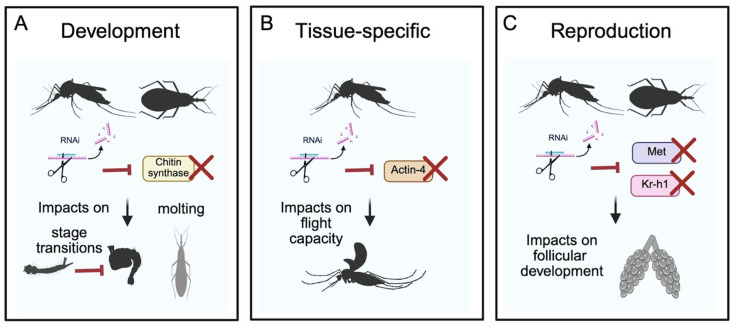
Potential genes for RNAi-based insecticides. (**A**) In mosquitoes, interference with the chitin synthase gene disrupts chitin biosynthesis, leading to disruptions in the larva-to-pupa transition. In triatomines, disruption of the chitin synthase gene causes morphological alterations due to impaired molting. (**B**) In mosquitoes, the expression of the actin-4 gene (act4) is sex-specific and begins at the larval stage; disruption of this gene results in flightless females. (**C**) Interference of genes related to Juvenile Hormone (JH) signaling, including Methoprene-tolerant (Met) and the zinc-finger transcription factor Krüppel homolog-1 (Kr-h1), reduces ovarian follicle size, disrupts egg morphology and egg laying, and causes restriction in ovarian development. A summary of reported silencing approaches in insect vectors targeting proposed genes associated with development, metabolism, and reproduction is provided in Table S1. Created in BioRender. Maya-Maldonado, K. (2025) under agreement number TN28TU5SZS.

In hematophagous insects, such as mosquitoes, the peritrophic matrix is formed in response to the blood feeding [31]. Thus, several proteins related to PM formation, including the chitin synthase gene, are overexpressed in response to the blood meal [32,33,34]. Interestingly, in *Culex pipiens*, the interference of the chitin synthase A (CHSA) gene results in gut permeability and a decrease in female reproduction [35,36]. Furthermore, such interference also impairs chitin biosynthesis during molting, thereby affecting the transition from larval to pupal stage. In *Rhodnius prolixus,* the injection of dsRNA targeting the chitin synthase gene failed to induce molting, resulting in morphological alterations of the body and arrest in development [37]. Thus, chitin synthase represents a potential target for a new bio-insecticide based on RNAi, with the capacity to be applied during larval stages to disrupt development in multiple vectors (Figure 2A and Table S1).

#### 3.1.2. Actin Silencing to Block Flight Capacity

Actin is a structural protein that forms part of the thin actin filaments (F-actin), necessary for the integration of the muscular contractile unit, the sarcomere. Through interaction with myosin, its purpose is to generate muscular contraction [38]. Once metamorphosis occurs during the pupal stage, myofilaments are organized into structures that shape the flight muscles. Myogenesis of flight muscles has been described in mosquitoes, where F-actin filaments play an essential role in sarcomere formation. A study of the fourth-instar larvae of *Ae. aegypti* shows that F-actin is initially organized into short filaments, where myosin is subsequently incorporated. These myofilaments, through cell fusion, form premyofibrils, precursors of the mature myofilaments of flight muscles [39]. Particularly, flight muscles require a high level of contraction. As such, F-actin is organized into finely structured networks that allow wing movement. In *Drosophila,* the act88F gene encodes an isoform of actin, and its principal characteristic is that it exhibits tissue-specific expression properties [40]. For instance, in the mosquito *Ae. aegypti*, the actin-4 gene (act4) is a paralog of act88F, and its expression is limited to the flight muscles of females since larval stages [41].

A study using the promoter region of the act88F gene from *Drosophila* identified this promoter as a suitable strategy to drive specific gene expression only in the flight muscles of mosquitoes [42]. This opened a new possibility to use the act4 gene as a target for genetic population control strategies. In fact, a study by Navarro-Payá et al. (2020) utilized the CRISPR/Cas9 gene editing system to delete the act4 gene in *Ae. aegypti* and *Culex quinquefasciatus*, they observed that short deletions of 6 bp produce, only in females, a flightless dominant phenotype, and that deletions by insertion of a cassette produce a recessive phenotype [43]. Furthermore, silencing this gene using dsRNA in *Anopheles albimanus* has been reported to produce flightless females and a significant mortality rate in both females and males, with a greater effect observed when the dsRNA includes the 3′ UTR and coding terminal domain regions [44]. These works demonstrate that the act4 gene is functional for genetic population control strategies aimed at eliminating female mosquitoes. One of the main advantages of using the act4 gene is that it is sex-specific, as it is primarily expressed in females from the larval stage onwards. This allows for a more selective and targeted approach, directly blocking the flight capacity of females, since they are the ones that, by feeding on blood, in the case of vector mosquitoes, disperse the pathogens, and are also unable to mate with males. Therefore, since there are flight muscle-specific F-actin genes in various arthropods, it becomes a strategic target for biological control through the use of RNAi (Figure 2B and Table S1).

Inhibition of actin gene expression has been achieved not only in mosquitoes but also in other insect orders. For example, in bed bugs (*Cimex lectularius* L.), the injection of dsRNA targeting actin shows a significant decrease in oviposition and survival [45]. In addition, in insect pest, inhibition of this gene results in developmental delay, and high mortality [46,47], illustrating successful examples of actin silencing in other insects that can be extrapolated to vectors. Specifically for mosquitoes, RNAi-based strategies that block the flight capacity offer the possibility of generating populations of flightless phenotypes. Moreover, due to their effects on vital functions, not only flight, but also muscle contraction, development, and reproduction, this strategy can help control vector populations in areas with a high prevalence of disease transmission.

### 3.2. Targeting Genes Related to Reproduction

Insects exhibit a high reproductive capacity, which significantly contributes to their success in invading and pathogen transmission. Due to this, targeted strategies to limit insect reproduction can be beneficial in combating vector-borne diseases. Reproductive physiology of insects is orchestrated for several processes, including Juvenile Hormone (JH) signaling [48]. As a gonadotropic hormone, JH stimulates insect vitellogenesis and oogenesis (for an overview in model organisms see [48]). As such, in mosquitoes, JH controls ovarian development by allowing the development of primary follicles in the ovaries after adult emergence [49,50]. Interestingly, after a blood meal, JH levels decrease, which is crucial for transitioning to a vitellogenic state [51] where yolk proteins are synthesized and deposited in the ovaries for development.

JH regulates the expression of several genes through the downstream action of the receptor, Methoprene-tolerant (Met). Importantly, in *Ae. aegypti* mosquitoes, the interference of Met and the zinc-finger transcription factor Krüppel homolog-1 (Kr-h1), a transcription factor regulated by Met, reduces the primary ovarian follicle size [52]. In other blood sucking insects, such as *R. prolixus*, JH signaling also regulates reproduction. For example, the silencing of Met restricts ovarian development [53]. A more recent study employed an RNAi strategy to knock down the expression of Met, the steroid receptor Taiman (Tai), and Kr-h1. The study indicates the role of Met and Tai in egg morphology, egg laying, and hatchability. In addition, a dramatic effect on ovarian development was observed during Tai silencing, indicating a critical role in reproduction [54]. Similarly, in another Triatomine, *Dipetalogaster maxima,* silencing of Met decreases the expression of the vitellogenin (VgR) and lipophorin (LpR) Receptor genes, which impairs vitellogenesis and reduces ovarian development [55].

The insulin pathway is another signaling pathway that influences insect reproduction. For example, in several insect species, silencing of the transcription factor *FoxO*, which is the leading actor downstream of the insulin signaling pathway, impacts negatively reproduction [56,57,58]. Similarly, reduced fecundity and impaired ovarian development were observed when *FoxO*, or genes targeted by *FoxO*, are silenced in mosquitoes of the *Aedes* and *Culex* species [59,60]. Altogether, these functional studies in mosquitoes, using an RNAi approach, reveal a significant contribution of Met and other genes related to JH signaling affecting reproduction. Notably, RNA interference technology, in combination with nanotechnology, has emerged as a sustainable strategy for agriculture. A successful example in Lepidopterans targeted *Met* expression during larval stages, where nanoliposome structures are used to protect and enhance dsRNA delivery reduced fitness traits such as pupal weight [61]. Similarly, Met and other JH signaling genes could be potential candidates for impairing the development and reproduction of insect vectors (Figure 2C and Table S1).

For years, JH signaling has been a target to control insect development. In fact, the JH analog methoprene has been widely used as an insecticide [62,63]. Additionally, methoprene has been used in combination with acaricides to control infestations by ectoparasites in cats and dogs [64,65,66]. Some studies claim the safe environmental use of methoprene [63]; however, its use has also raised concerns and generated debatable opinions about potential adverse effects in non-targeted species [67,68]. Thus, non-chemical insecticides, such as RNAi-based insecticides, offer an alternative to chemical insecticides, providing advantages over their chemical counterparts, including high specificity and limited side effects to non-target species.

In addition, these studies also indicate that the downstream genes of the JH and insulin pathways are remarkably conserved among insects, underscoring their potential as targets for RNAi-based gene silencing strategies. However, the conservation of these proteins between insect species could be a significant concern when selecting them as candidates for generating new-generation RNAi-based insecticides. In fact, one of the most essential characteristics for successful insect vector intervention strategies is specificity. In this sense, RNAi-based insecticides offer the possibility of silencing a gene of interest in a specific and efficient manner through the delivery of dsRNA.

Another strategy is the selection of genes with time-specific or inducible expression. Principally, insect vectors rely on a blood meal to initiate a gonadotropic cycle. Thus, proteins regulated only in response to blood feeding and associated with reproduction are potential candidates for vector intervention strategies. In fact, a recent work in *Aedes aegypti* characterized a cysteine-rich trypsin inhibitor-like protein, named cysteine-rich venom protein 379 (CRVP379), which is strongly expressed in ovaries and testes. The authors also demonstrated that mutations in CRVP379 result in the abnormal morphology of follicular cells in *Ae. aegypti* ovaries, indicating a role in mosquito reproduction [69]. This study offers an additional candidate for designing next-generation insecticidal RNAi formulations.

## 4. Advances and Challenges in dsRNA Delivery in Vectors

Agricultural pest management using RNAi-based pesticides has seen significant advances in translational research, current applications, and commercialization. A key factor contributing to this progress is that many aspects of crop pests’ life cycles are closely tied to their host plants [70]. This close association facilitates the targeted control of insect pests and the protection of host plants through various pest management strategies, including RNAi-based pesticides.

In contrast, insect vectors, such as mosquitoes, have more complex life cycles. Mosquitoes are born in aquatic environments and undergo four larval stages before becoming pupae (a non-feeding stage). Upon emergence as adults, males and females exhibit different feeding behaviors: males feed exclusively on nectar, while females feed on both nectar and blood. The blood meal is essential for egg production and completing the life cycle [71]. Furthermore, some mosquito species preferentially feed on humans, while others also feed on other animals [72]. Mosquitoes are just one example, but other vectors, such as ticks, sand flies, kissing bugs, etc., exhibit different behaviors and inhabit distinct environments, requiring tailored strategies for effective population control [73], including the use of RNAi-based pesticides.

Since the first report of using dsRNA for gene silencing in mosquitoes and ticks in 2002 [74,75] and in kissing bugs in 2006 [76], RNAi has been an invaluable tool for studying gene function in vector biology research. In these studies, dsRNA has typically been delivered by injection, because the experiments involve the use of a few specimens; however, for the deployment of dsRNA-based biopesticides, alternative strategies have been employed to target multiple life stages of several vector species. In the section below, the approaches already used for the delivery of dsRNA with potential for field deployment are discussed.

### 4.1. Delivery Approaches

#### 4.1.1. Soaking and Oral Feeding (Naked dsRNA)

The simplest method to deliver dsRNA is through soaking, which involves immersing or feeding the organism in a solution containing dsRNA, typically water or saline solution. This approach has been effective under laboratory conditions in mosquitoes [77,78,79,80], ticks [81], and kissing bugs [76]. However, in natural environments, only mosquitoes possess aquatic life stages, and soaking is not a practical delivery method for ticks and kissing bugs in the wild. Although gene silencing using naked dsRNA has primarily been demonstrated in the larval stages of *Aedes* and *Culex* mosquitoes, this approach requires higher concentrations of dsRNA compared to injection [78,79,80]. Additionally, naked dsRNA is highly susceptible to degradation by environmental and gut vector nucleases, which significantly reduces its efficiency [82]. Due to its several disadvantages, delivering naked dsRNA is not considered a viable approach for RNAi-based biopesticide applications.

#### 4.1.2. Nanoparticles

Since dsRNA is highly susceptible to degradation, several strategies have been developed to protect it, including the use of nanoparticles as carriers. This approach has seen significant advances in the field of agriculture pest management, as nanoparticles offer controlled release, enhanced stability, and higher specificity [83]. Nanoparticles as a dsRNA delivery system have been made from different materials, including but not limited to chitosan, lipids (Liposomes), carbon, silica, and clay minerals [83], but only a few of these have been tested for delivery in vectors.

In mosquito larvae, chitosan, carbon quantum dots, and silica nanoparticles have been successfully used to deliver dsRNAs and siRNAs due to their high stability in water [84,85,86,87,88,89]; however, this method has not yet been tested in other insect vectors. In *Cimex lectularis*, the administration of chitosan-silver particles by soaking resulted in high mortality rates, suggesting that they may similarly target ticks and kissing bugs [90], and most likely function as dsRNA carriers as well; however, future studies are required to address this hypothesis.

#### 4.1.3. Liposomes

Liposomes are considered one of the most promising drug delivery systems, including those based on nucleic acids [91]. The latest liposomes used in pest management offer protection to dsRNA from environmental stressors such as high temperatures, RNases degradation, and UV radiation [92]. In *Ae. aegypti* larvae, ingestion of dsRNA encapsulated in liposomes leads to gene silencing [93]. Ticks, *Rhipicephalus haemaphysaloides*, soaked in liposomes containing dsRNA, have also shown efficient gene silencing [94]. Although in kissing bugs liposomes has not been tested, most likely has similar efficiency. Although liposomes have shown high efficiency in delivering dsRNA, they are expensive and less stable compared to other methods.

#### 4.1.4. Microorganisms Delivery Systems

##### Genetically Modified Microorganisms (Bacteria, Yeast and Alga)

Live microorganisms have been more widely used to deliver dsRNA in several vector species. This is because live microorganisms can partially colonize the insect’s body and its environment, thereby minimizing the time needed to release these biopesticides and the cost.

Most studies employ *Escherichia coli* HT115 (DE3), a strain deficient in RNase III activity and possessing T7 RNA polymerase, both of which contribute to enhanced dsRNA synthesis [95,96]. This strain has been used to deliver dsRNA in mosquitoes *Ae. aegypti* [77,80,82], and *R. prolixus* [97], which efficiently result in the silencing of several genes. Several common laboratory *Saccharomyces cerevisiae* strains have also been used to induce gene silencing by delivering dsRNA or short hairpin RNAs (shRNAs) in mosquitoes *Ae. aegypti* [98,99]. 

Another interesting strategy, which targets mosquito larvae due to their feeding behavior, involves the use of genetically modified microalgae. Since microalgae are one of the primary food sources for mosquito larvae [100,101], they are a promising vehicle for delivering larvicidal agents. The *Chlamydomonas* and *Chlorella* microalgae have been used as dsRNA and shRNA delivery systems to induce gene silencing in *Ae. aegypti*, *Ae. albopictus* and *An. stephensi* [102,103,104,105]. While this strategy has started to be tested in mosquitoes and kissing bugs, it has yet to be explored in ticks. Additionally, since it involves the use of genetically modified microorganisms, it requires stringent biosafety regulations, which can delay its application in the field. However, it still represents a cost-effective strategy with great potential for field applications.

##### Heat-Killed Genetically Modified Microorganisms (Bacteria and Yeast)

Although chemical encapsulation using chitosan, lipids, or other materials is a feasible strategy for protecting and delivering dsRNA, its effectiveness depends on the sustained release of dsRNA and its ingestion by the target insect, which significantly increases the cost [106]. On the other hand, releasing genetically modified microorganisms into the environment requires increased biosafety studies and government approval, which could delay their implementation.

To address this issue, several studies have employed inactivated genetically modified microorganisms such as bacteria and yeast that express dsRNA. Heat-killed genetically modified *E. coli* expressing dsRNA have been employed to induce gene silencing in larvae of *Ae. aegypti*, *An. gambiae* [77,80,89,98,107] and the salivary glands of adult *An. gambiae* mosquitoes [108]. Additionally, heat-killed genetically modified yeast that express shRNAs or siRNAs have successfully induced gene silencing in several mosquito species, including *An. gambiae*, *Ae. aegypti*, *Ae. albopictus*, and *Culex quinquefasiatus* [99,107,109,110]. This strategy has largely been used in laboratory trials on mosquitoes; however, it remains unexplored in ticks and kissing bugs.

##### Paratransgenesis

Paratransgenesis refers to the use of genetically modified symbionts to deliver recombinant proteins and peptides, to disrupt host–pathogen interactions, reproduction, and other processes without modifying the genome of the target organism [111]. Genetically modified microorganisms such as symbionts offer several advantages over other delivery methods. They can stably colonize insect hosts and be transmitted both horizontally and vertically, enabling long-term and sustained effects [111,112].

In *R. prolixus*, genetically modified symbionts expressing dsRNA have been used to silence the vitellogenin gene expressed in the fat body [113], the Rhodnius heme-binding protein (RHBP) in eggs, and catalase in all tissues [97]. In the mosquito *An. stephensi*, the same strategy has been used to silence the methoprene-tolerant gene and ecdysone receptor [114]. While paratransgenesis has been employed in ticks to deliver peptides and proteins, the use of this tool for dsRNA delivery remains unexplored and likely represents a promising approach to gene silencing in these vectors.

### 4.2. Advances and Limitations of dsRNA Delivery for Targeting Insect Vectors in the Field

Most delivery systems have been directly tested in the field and have shown high efficiency in crop pest management. However, an outstanding question that remains is why the success of RNAi-based insecticides in agriculture has not yet been translated for vector control. In agriculture these strategies are often applied directly to the insects or through foliar spraying, enabling dsRNA ingestion during feeding [10]. Nevertheless, vectors are blood-sucking insects, which, in the case of kissing bugs and ticks, feed exclusively on the blood of vertebrates, which discards the use of agriculture methods for releasing these bioinsecticides.

In addition, insects exhibit differences in the efficiency of gene silencing when applying dsRNA, which depend on the species, method of application (injection, feeding, or soaking), target gene, and the concentration of the dsRNA used [115]. Unfortunately, diptera and hemiptera insects, orders that mosquitoes and kissing bugs belong to, have shown poor efficiency in gene silencing, and high concentrations of dsRNA between 1 and 80 μg are required to achieve similar effects to coleoptera, which only require around 50 ng to induce efficient gene silencing (See detailed information in Hanneke Huvenne, Guy Smagghe, 2010 [115]). The requirement of high concentrations of dsRNA has several implications, as increased dsRNA synthesis leads to higher production costs, and the encapsulation of a large amount of dsRNA to avoid degradation is technically challenging.

It is important to note that the delivery approaches previously tested in vectors (summarized in Table 1) are mainly applied under laboratory conditions, where insects are induced to feed on dsRNA, allowing controlled concentrations and preserving its integrity. However, these delivery approaches for field trials have been unexplored. In the field, the habitats and behavior of vectors are not controlled, and environmental conditions compromise the optimal concentration and integrity of dsRNA required for silencing. These conditions have been the main challenge for the field application of dsRNAi-based insecticides for controlling insect vectors. In the following section, we discuss some strategies currently being tested on specific vectors, as well as others that could be extrapolated to them.

#### Potential Strategies for Field Delivery of dsRNA Targeting Vectors

In agriculture, RNAi approaches are applied directly to insects or through foliar application, allowing insects to ingest the dsRNA when they feed on the treated plants [10]. Nevertheless, vectors are blood-sucking insects that exhibit different behaviors [116], which requires the development of new dsRNA delivery strategies that consider this features of each vector.

**Figure 3:**
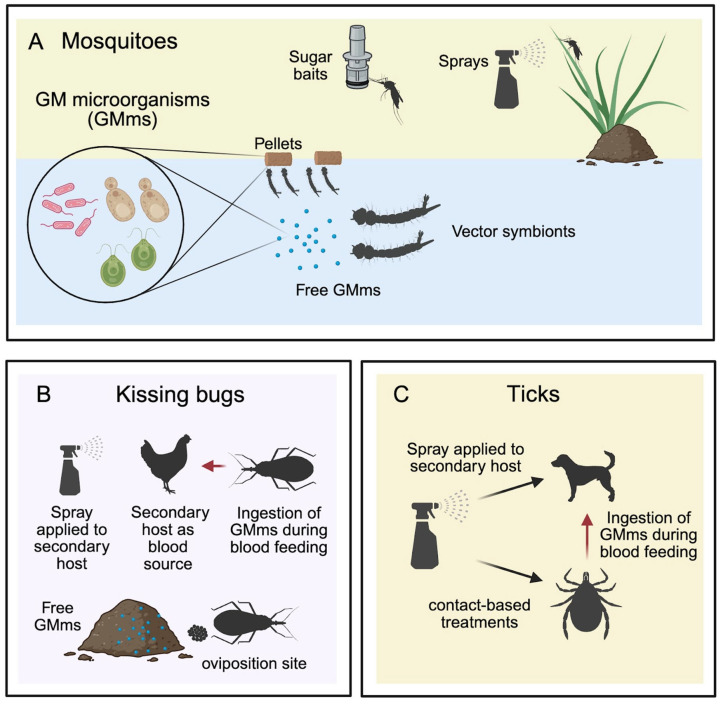
Potential strategies to deliver dsRNA into insect vectors. (**A**) In mosquitoes, Genetically Modified microorganisms (GMms), including algae, bacteria, or yeast, could be administered to larvae in pellets or in free form, released directly into water sources, where they could become symbionts of the vectors. Similarly, microorganisms expressing dsRNA (GMms) can be deposited on attractive target sugar baits. In addition, a potential strategy would be the direct application of sprayable RNAi-based (dsRNA) insecticides. (**B**) For kissing bugs, secondary hosts can be used as vehicles for dsRNA delivery. A spray containing microorganisms expressing dsRNA can be applied to a secondary host, which will then be used as a food source for vectors. Vectors would be able to acquire, for example, bacteria expressing dsRNA to target specific genes. In addition, free GMms can be delivered to potential oviposition sites, where a new generation of vectors can come into direct contact with the microorganisms expressing dsRNA. (**C**) For ticks, the application of spray to a secondary host could be used, as in kissing bugs. In addition, a contact-based treatment, including direct application of sprayable RNAi-based (dsRNA) insecticides, might also be employed on infested animals. Created in BioRender. Maya-Maldonado, K. (2025) under agreement number YE28TU66J8.

Since only adult female mosquitos need to feed on blood, larval stages are typically the target for releasing RNAi-based pesticides. Genetically modified algae, bacteria, yeast, or pellets containing these dead/living microorganisms, as well as nanoparticles containing dsRNA are deposited in water breeding [89,98,99,103,104,105,107,109,110,114], where mosquito larvae fed on them. Because female and male adult mosquitoes also feed on nectar, the use of attractive target sugar bait containing, for example, genetically modified yeast expressing dsRNA has been tested to target adult stages [109] (Figure 3A). Because microorganisms such as *S. cerevisiae* or *E. coli*, nanoparticles, and liposomes are not typically part of the diet of these insects, these strategies aim to reach the larval or adult stages by getting attracted depending on their feeding behaviors. When combining dsRNA delivery/encapsulation methods with attractive food for mosquitoes, we ensure they ingest the dsRNA while minimizing ingestion by non-target species. However, the combined approaches have been tested under laboratory conditions, and semi-field trials are required to demonstrate their efficiency.

Ticks have a four-stage life cycle: egg, larva, nymph, and adult. Tick eggs are usually laid in the soil, grass, or leaf litter, where they hatch into six-legged larvae. These larvae seek out a host to feed on and then molt into the nymph stage. Nymphs also feed on one or more hosts before molting again into the adult stage [117]. On the other hand, kissing bugs have a three-stage life cycle: egg, nymph, and adult. To reach adulthood, each nymphal stage requires a blood meal. Interestingly, kissing bugs exhibit nocturnal feeding behavior and feed on various hosts, primarily birds and mammals [118].

Both ticks and kissing bugs pose challenges for the application of insecticides, including those based on RNA interference (RNAi). Nevertheless, a common factor among these vectors is their tendency to feed on secondary hosts that are not humans. A recent study explored the use of rodents as vehicles to introduce genetically modified symbiotic bacteria from the vector. In this study, rodents were fed pellets containing live bacteria. These bacteria were able to cross the intestinal tract and were subsequently excreted in the rodents’ feces. Sand fly larvae then feed on this feces, allowing the modified bacteria to be transmitted in a manner that persists through various life stages of the insect [119]. A similar strategy could be employed to introduce genetically modified symbionts into ticks and kissing bugs, which typically feed on domestic animals like dogs, cows, cats, and chickens in areas inhabited by humans. Releasing these symbionts on secondary hosts or within vector habitats would enable the vectors to acquire the bacteria through natural contact, as they are highly exposed during feeding. This strategy could significantly impact kissing bugs, as they typically hide in dark areas, making their habitats challenging to identify for effective insecticide application (Figure 3B).

Another delivery strategy to target ticks and mosquito adults is by topical applications. Ticks are highly exposed when they infest animals, and mosquitoes often rest on surfaces both indoors and outdoors, making them accessible to contact-based treatments such as spray. In aphids, the delivery of dsRNA through topical applications has demonstrated effective gene silencing and resulted in high mortality rates [120,121]. This suggests that employing a similar strategy could yield comparable outcomes in insect vectors like ticks and mosquitoes (Figure 3C).

Although these strategies have shown potential in other vectors and crop pests, such as sandflies and aphids, their effectiveness on mosquitoes, ticks, and kissing bugs should be tested first under laboratory conditions, followed by semi-field trials. Future studies are necessary to apply these strategies for controlling vector populations.

## 5. dsRNA-Based Insecticides: Advantages in Specificity over Conventional Pesticides

Conventional insecticides, including synthetic or those derived from natural compounds found in plants or microorganisms, have been used for a long time to control insect populations. However, their specificity is very low and seriously affects non-target species such as pollinators. Such non-specificity is a double-edged sword, as it targets diverse insect species, which significantly reduces production costs; however, it also affects other beneficial insect species and may have adverse effects on human health [122]. Most of the insecticides target enzymes and proteins related to the nervous system, metabolism, and growth regulation [122,123]. The primary issue or advantage with these insecticides is that their target sites are highly conserved in structure and function across various taxonomic levels, including genus, family, order, class, and phylum. This conservation is why these insecticides have a broad spectrum of activity against multiple insect species [122,124].

A good example of this is the methoprene, which mimics the juvenile hormone (JH) of insects, and its application disrupts the molting and morphogenesis of larval stages of several insect species, including mosquito larvae [63,125]. Although it has effectively reduced mosquito populations, its use in many countries has been restricted due to concerns about its effects on non-target species [63] The JH pathway is highly conserved among invertebrates; however, the genes that code for the proteins in this pathway are not the same, and even the conserved ones do not have similar sequences [126]. By using RNAi approaches to target these genes (see Section 3), we can reduce the risk of affecting non-target species while achieving effects similar to those of JH analogs, such as methoprene.

The specificity of gene silencing using dsRNA is extremely high. In fact, a pioneering study of dsRNAi-based insecticides revealed that four closed species of coleopterans exhibit different susceptibilities to gene silencing, even when targeting the same gene [11]. On the other hand, siRNAs produced by dsRNA are small RNA molecules that are 21–22 nucleotides long. Due to the probabilistic nature of the 4-nucleotide combination, there is a chance these siRNAs could inadvertently target genes in both the non-target genes of the target species and non-target species. However, rapid advancements in bioinformatics and genomic approaches have made it possible to design dsRNA sequences that produce siRNA with no matches in other species, thereby minimizing the risk of cross-reactivity [127].

## 6. Perspectives of RNAi-Based Insecticides to Control Insect Vectors

Insect vectors have spread around the world, mosquitoes and ticks are present in all the continents except for the Antarctic [128,129], and kissing bugs have spread throughout the Americas and cause great concern because of the adaptation of the vectors [130]. These vectors transmit several pathogens, for which there are currently no effective vaccines, and in the case of arboviral infections, there are no drugs. Due to this, the primary strategy for controlling vector-borne diseases has been the use of insecticides. Currently, a global strategy for large-scale insecticide application includes Indoor Residual Spraying (IRS). According to the WHO, IRS involves the application of insecticides in potential resting sites for vectors, with the aim of killing the vectors to limit the transmission of pathogens to humans. Historically, the IRS strategy has been a powerful intervention to reduce the number of mosquitoes in areas with high insect vector densities. However, the use of insecticides inherently poses risks to humans, as well as concerns about ecological and environmental impacts [131].

Other strategies that are considered ecofriendly, such as Sterile Insect Technology (SIT), have been used to control pests like the screwworm and medfly [132]. However, a similar strategy that was used to control mosquito populations was unsuccessful due to several factors, including their widespread distribution, complex life cycle, the need for constant release of sterile insects, sex sorting challenges, and other behavioral characteristics [132,133]. In recent years, the development of genetic tools, such as the CRISPR-based gene drive system, has emerged as a promising SIT. This approach offers sustainable population suppression, since over time, the modified insects can replace the wild population [133]. Both strategies face several challenges. They require a long time to show results, their modes of action are limited to small areas, the strategy itself is limited to one species at a time, and wild populations may resist them due to mating behaviors. Additionally, genetically modified insects necessitate further biosafety studies, which will also take time [134]. In contrast, RNAi-based insecticides exhibit similar effects, also based on genetic manipulation, without the drawbacks associated with the generation and release of genetically modified organisms.

In summary, this review discusses why RNAi-based insecticides have become applicable in agriculture, with the first commercially available products already on the market. Meanwhile, studies on insect vectors of human diseases have mostly been limited to laboratory trials. Although the field application of RNAi-based insecticides for insect vector control faces several challenges, their success in agriculture offers valuable lessons (discussed on Section 2), such as delivery approaches, target selection, and biosafety regulations, which will hopefully accelerate the process of field application. RNAi-based insecticides have limitations and advantages; however, they represent an ecological alternative to conventional insecticides, which have been historically used to successfully suppress insect populations such as crop pests and insect vectors.

## Figures and Tables

**Table 1:** Advantages and disadvantages in delivery approaches for insect vectors.

Delivery Approaches	Tested on (Insects) ^b^	Advantages	Disadvantages	References
Injection	Mosquitoes, Ticks, Kissing bugs	High silencing efficiency	Limited scalability, research use only	[74,75,76]
Soaking and feeding/oral delivery	Mosquitoes, Ticks, Kissing bugs	Simple application	Low environmental stability, research use only	[76,77,78,79,80,81,82]
Nanoparticles	Mosquitoes, Ticks	High stability	Expensive	[84,85,86,87,88,89]
Liposomes	Mosquitoes, Ticks	High silencing efficiency	Low environmental stability, costly	[93,94]
GMms ^a^ Alive	Mosquitoes, Kissing bugs	Low cost, high stability	GMO regulations, constant release	[77,80,82,97,98,99,102,103,104,105]
GMms ^a^ Heat-killed	Mosquitoes	Low cost, high stability	Constant release required	[77,80,89,98,99,107,108,109,110]
GMms ^a^ Symbionts (paratransgenesis)	Mosquitoes, Kissing bugs	Low cost, high stability	GMO regulations	[97,113,114]

^a^ Genetically modified Microorganisms. ^b^ Trials under laboratory conditions.

## Data Availability

Not applicable.

## Supplementary Materials

The following supporting information can be downloaded at: https://www.mdpi.com/article/10.3390/genes16111276/s1, Table S1: Summary of reported silencing approaches in insect vectors targeting proposed genes associated to development, metabolism, and reproduction.
