# Supplementary material for: RNAi-Based Bioinsecticides for Controlling Vector-Borne Diseases
Source: Genes (Basel). 2025 Oct 28;16(11):1276. doi: 10.3390/genes16111276 (PMC12652134; doi:10.3390/genes16111276)
Supplement: Supplementary file 1 [file genes-16-01276-s001.zip › genes-3852548-supplementary.pdf]

Table S1. Summary of reported silencing approaches in insect vectors targeting proposed genes associated to development, metabolism, and reproduction.

Insect Order	Insect Specie	Target gene	Delivery method/approach	Phenotypes	References
Diptera	*Culex pipiens*	Chitin synthase A (*CHSA*)	small interfering RNA (siRNA) injection	gut permeability, reduced female reproduction, impaired larva–pupa molting	Wang et al., 2023 [35], Yang et al., 2021 [36]
Hemiptera	*Rhodnius prolixus*	Chitin synthase (*CHS*)	dsRNA injection	failure to molt, morphological alterations, arrested development	Mansur et al., 2014 [37]
Diptera	*Ae. aegypti* and *Culex quinquefasciatus*	Actin (*act4*)	Gene knockout (CRISPR/Cas9)	female-specific flightless phenotype	Navarro-Payá et al., 2020 [43]
Diptera	*An. albimanus*	Actin (*act4*)	Oral delivery of dsRNA	flightless females, high mortality (males and females)	Paiz-Reyes et al., 2020 [44]
Hemiptera	*Cimex lectularius L*	*Actin*	dsRNA injection	decreased oviposition and survival	Basnet and Kamble, 2018 [45]
Diptera	*Ae. aegypti*	Methoprene-tolerant (*Met)* and zinc-finger transcription factor Krüppel homolog-1 (*Kr-h1)*	dsRNA injection	reduced ovarian follicle size	Saha et al., 2019 [52]
Hemiptera	*Rhodnius prolixus*	*Met*	dsRNA injection	restricted ovarian development	Villalobos-Sambucaro et al., 2015 [53]
Hemiptera	*Rhodnius prolixus*	Steroid Receptor Taiman (*Tai)*	dsRNA injection	abnormal egg morphology, reduced egg laying, and impaired hatchability	Leyria et al., 2022 [54]
Hemiptera	*Dipetalogaster maxima*	*Met*	dsRNA injection	reduced VgR/LpR expression, impaired vitellogenesis, decreased ovarian development	Ramos et al., 2022 [55]
Diptera	*Ae. aegypti*	Forkhead transcription factor (*FoxO*)	dsRNA injection	reduced fecundity	Hansen et al., 2007 [59]
Diptera	*Culex pipiens*	*FoxO* and Insulin Receptor (*InR*)	dsRNA injection	impaired ovarian development, reduced follicle length	Sim and Denlinger, 2008 [60]
Diptera	*Ae. aegypti*	Cysteine-rich venom protein 379 (*CRVP379*)	dsRNA injection and Gene	abnormal ovarian follicular cell morphology, impaired reproduction	Tikhe et al., 2022 [69]

			knockout (CRISPR/Cas9)		
